# Altered High Density Lipoprotein Composition in Behavioral Variant Frontotemporal Dementia

**DOI:** 10.3389/fnins.2018.00847

**Published:** 2018-11-14

**Authors:** Woojin Scott Kim, Ying He, Katherine Phan, Rebekah M. Ahmed, Kerry-Anne Rye, Olivier Piguet, John R. Hodges, Glenda M. Halliday

**Affiliations:** ^1^Brain and Mind Centre, The University of Sydney, Sydney, NSW, Australia; ^2^School of Medical Sciences, University of New South Wales, Sydney, NSW, Australia; ^3^Neuroscience Research Australia, Sydney, NSW, Australia; ^4^ARC Centre of Excellence in Cognition and its Disorders, Sydney, NSW, Australia; ^5^School of Psychology, The University of Sydney, Sydney, NSW, Australia

**Keywords:** frontotemporal dementia, Alzheimer’s disease, HDL, LDL, apolipoprotein, lipid, biomarker, cardiovascular disease risk

## Abstract

Frontotemporal dementia (FTD) is a common cause of early onset dementia with behavioral variant FTD (bvFTD) being the most common form. bvFTD is characterized clinically by behavioral and personality changes, eating abnormalities, and pathologically, by systemic lipid dysregulation that impacts on survival. As lipoprotein metabolism is at the core of lipid dysregulation, here, we analyzed the composition, both proteins and lipids, of the two major lipoprotein classes in blood – high density lipoproteins (HDLs) and low density lipoproteins (LDLs). Fasted plasmas from bvFTD and Alzheimer’s disease (AD) patients and controls were fractionated using fast protein liquid chromatography (FPLC) and samples analyzed by lipid assays, ELISA and western blotting. We found that apolipoprotein A-I (apoA-I) and apolipoprotein A-II (apoA-II) levels in HDLs were decreased in bvFTD compared to controls, whereas apolipoprotein B (apoB) levels in LDLs were unaltered. We also found that cholesterol and triglyceride levels in FPLC fractions were altered in bvFTD compared to controls. The apoB:apoA-I ratio and the standard lipid ratios were significantly increased in bvFTD compared to AD and controls. Furthermore, we found that plasma apolipoprotein C-I and paraoxonase 1 levels were significantly altered in bvFTD and AD, respectively, compared controls. This study represents the first apolipoprotein analysis of bvFTD, and our results suggest altered HDL function and elevated cardiovascular disease risk in bvFTD.

## Introduction

Proper diagnosis and treatment of dementia is a major challenge to public health with the aging of the population. Two major causes of dementia are Alzheimer’s disease (AD) and frontotemporal dementia (FTD), with behavioral variant FTD (bvFTD) the most common form of FTD (Piguet et al., 2011). Pathologically, bvFTD and AD are distinctive proteinopathies. Unlike AD, bvFTD is characterized by considerable early loss of significant amounts of brain tissue with concomitant loss of lipids (Broe et al., 2003; Kril and Halliday, 2004; Kril et al., 2005; Gregory et al., 2006). However, bvFTD patients are often misdiagnosed as AD because of overlapping clinical traits. A key feature that differentiates bvFTD patients from AD patients is the presence of eating abnormalities in bvFTD that leads to increased body mass index and altered blood lipids (Ikeda et al., 2002; Rascovsky et al., 2011; Ahmed et al., 2014a,b; Kim et al., 2018).

Increasing evidence indicates a strong association between lipid metabolism and dementia, particularly in AD, with changes in blood lipid levels and distribution contributing to disease processes (Hottman et al., 2014). A central player in lipid metabolism is high density lipoproteins (HDLs). HDLs consist of populations of spherical particles of varying size that comprise a surface monolayer of phospholipids, mainly phosphatidylcholine, and apolipoproteins that surrounds a core of neutral lipids, primarily cholesteryl esters, and to a lesser extent triglycerides. Most of the triglycerides in blood are transported by very low density lipoproteins (VLDLs). Apolipoprotein A-I (apoA-I) and apolipoprotein A-II (apoA-II) make up approximately 70 and 20%, respectively, of HDL protein mass, and play important roles in structure and function of HDL (Shah et al., 2013). Other proteins associated with HDLs that could be relevant to HDL metabolism and/or dementia include apolipoprotein C-I, paraoxonase 1 (PON1), and serpin family A member 1 (SERPINA1).

High density lipoproteins are extremely dynamic and undergo constant remodeling and recycling. A primary function of HDLs is to carry excess cholesterol from peripheral cells to the liver, where it is converted to bile acids and excreted from the body, a process known as reverse cholesterol transport. HDLs have been extensively studied in the context of cardiovascular disease (CVD), and are commonly referred to as “good cholesterol” because of their anti-atherogenic properties. Increased plasma levels of HDL-cholesterol are strongly associated with a reduced risk of developing CVD (Miller and Miller, 1975). The two commonly used lipid ratios for determining CVD risk are triglyceride:HDL-cholesterol and total cholesterol:HDL-cholesterol.

The pathological link between apolipoproteins and bvFTD is unknown. Here, we analyzed the major apolipoproteins, as well as other proteins, in HDLs and LDLs in bvFTD, AD, and controls without neurological or psychiatric disorders. The aims of our study were to determine biochemical changes in lipoproteins in bvFTD compared to AD and controls, and identify biomolecules that could be considered for biomarker development for bvFTD. Our study sheds new light on understanding lipid dysregulation underlying bvFTD.

## Materials and Methods

### Patient Blood Collection

Patients with bvFTD (M/F: 4/6), patients with AD (5/5), and healthy controls (5/6) were recruited at Neuroscience Research Australia in Sydney from FRONTIER, the FTD clinical research group, and from a panel of healthy study volunteers (Ahmed et al., 2014b) with no neurological (i.e., no evidence of cognitive impairment) or psychiatric disorders. The study was approved by the University of New South Wales human ethics committee (approval number: HC12573). Blood samples were obtained following written informed consent from the participant and/or primary carer. All patients underwent a comprehensive neurologic and cognitive assessment (e.g., clinical interview, neurologic examination, cognitive assessment, and structural brain MRI) to meet their respective diagnostic criteria (McKhann, 2011; Rascovsky et al., 2011), as previously described (Ahmed et al., 2014b). The mean age of the three groups were 64.1 ± 7.9, 67.9 ± 8.4, and 70.7 ± 3.1 years, respectively. Fasted blood samples (4 mL) were collected in the morning following a 10-h fast in tubes (BD PST II Plastic Plasma Separator tube #367375), and plasma prepared by centrifugation at 3,500 rpm for 10 min at 4°C, which was then aliquoted and stored at −80°C until use.

### Fast Protein Liquid Chromatography (FPLC) and Assays

Frozen plasmas were thawed at room temperature and centrifuged at 13,200 rpm for 5 min at room temperature. Supernatants were filtered using 0.22 μM syringe filters and centrifuged as before. Samples (210 μL) were fractionated using an AKTA FPLC system (GE Healthcare Life Sciences) and two Superdex 200 Increase 10/300 GL columns connected in series. The samples were eluted with phosphate buffered saline (PBS) at a constant flow of 250 μL/min and 250 μL fractions were collected every min for 140 min. The process was monitored with the inbuilt spectrophotometer at an optical density of 280 nm. Fractions were analyzed using an AU480 Chemistry Analyzer (Beckman Coulter) to determine the concentrations of cholesterol, triglyceride, protein, apoA-I, apoA-II, and apoB as previously described (Stahler et al., 1977; Smith et al., 1985; Eugui et al., 1994). Phosphatidylcholine (the most abundant phospholipid in HDLs and LDLs) was assayed as previously described (Takayama et al., 1977).

### Western Blotting and ELISA

Equal volumes of fasted plasmas were separated on SDS–PAGE gels and transferred onto 0.2 μM nitrocellulose membranes at 75 volts for 30 min. Membranes were blocked with Tris buffered saline (TBS) containing 5% non-fat dry milk and probed with anti-apoAI antibody (rabbit polyclonal 1:5,000 Merck #178422) and anti-apoC-I antibody (rabbit monoclonal 1:1,000 Abcam #ab198288) overnight at 4°C. They were then washed three times in TBS containing 0.1% Tween 20 and incubated with horseradish peroxidase-conjugated secondary antibody for 2 h at room temperature. Signals were detected using enhanced chemiluminescence and Gel Doc System (Bio-Rad). The signal intensity was quantified using Image Lab (Bio-Rad) and NIH ImageJ software (v1.45s). Plasma PON1 (Cloud-Clone, #SEA243Hu) and SERPINA1 (Abcam, #ab108799) were measured by ELISA following the manufacturer’s protocols.

### Statistics

Statistical analyses were performed using SPSS Statistics software (IBM, Chicago, IL, United States). Multivariate analyses (general linear model) covarying for age and gender were used to determine differences in lipid and protein levels in the control, bvFTD and AD groups with *post hoc* statistical significance set at *p* < 0.05. Pearson correlations were used to determine if changes in apolipoprotein levels were associated with changes in lipid levels with statistical significance set at *p* < 0.05.

## Results

### Lipoprotein Profiles of bvFTD and AD Plasmas

**FIGURE 1:**
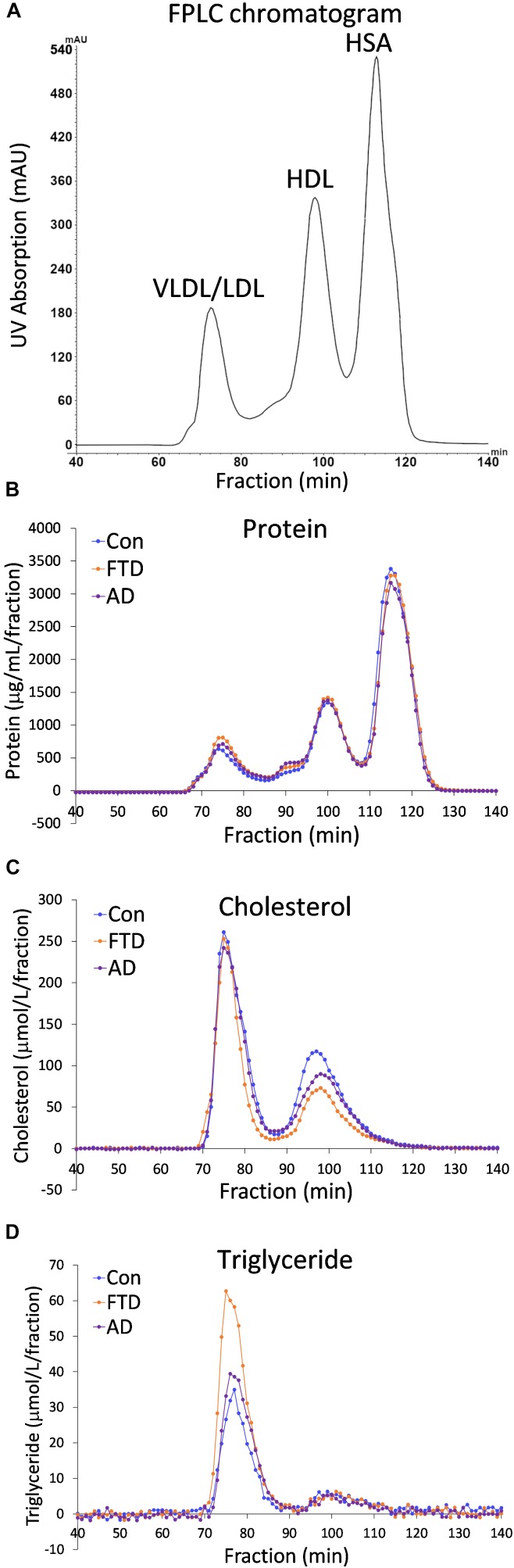
Fast protein liquid chromatography (FPLC) profiles of behavioral variant frontotemporal dementia (bvFTD), Alzheimer’s disease (AD), and control plasmas. **(A)** A typical FPLC chromatogram shows peaks at ∼75, 100 and 115 min that correspond to very low density lipoproteins/low density lipoproteins (VLDLs/LDLs), high density lipoproteins (HDLs), and human serum albumin (HSA), respectively. **(B)** FPLC protein profiles. **(C)** FPLC cholesterol profiles. **(D)** FPLC triglyceride profiles.

Despite the fact that dyslipidemia underlies bvFTD very little is known about biochemical changes in lipoproteins in bvFTD. Previous studies have utilized whole plasma to analyze differences in lipid levels in bvFTD compared to AD and controls (Ahmed et al., 2014b; Kim et al., 2018). Here, for the first time, we fractionated plasmas and analyzed apolipoproteins and lipids in the two major lipoprotein classes in blood – HDLs and LDLs. Fasted plasmas from bvFTD and AD patients and controls (*n* = 10, 10, 11) were individually fractionated using FPLC and 140 samples were collected over a 140-min period; in total 4,340 samples were collected from 31 plasma samples. A typical FPLC chromatogram generated from the inbuilt UV absorption detector revealed three peaks at ∼75, 100 and 115 min that correspond to VLDLs/LDLs, HDLs, and human serum albumin (HSA), respectively (Figure 1A). The concentrations of protein, cholesterol and triglyceride in the collected fractions were assayed and their profiles generated. The protein profiles showed three peaks, similar to the FPLC chromatogram, corresponding to VLDLs/LDLs, HDLs and HSA (Figure 1B). The cholesterol profiles showed two peaks corresponding to VLDLs/LDLs and HDLs (Figure 1C). The triglyceride profiles showed one large peak which is presumably VLDLs, and a very small peak that eluted at the position of HDLs (Figure 1D). These results confirm that the first peak is a mixture of triglyceride-rich VLDLs and LDLs, which contain cholesteryl esters and the second peak is HDLs which contain predominantly cholesterol and a small amount of triglyceride. The third peak is HSA which does not “carry” any cholesterol or triglyceride.

### Assessment of Apolipoproteins Carried by Lipoproteins in bvFTD and AD

**FIGURE 2:**
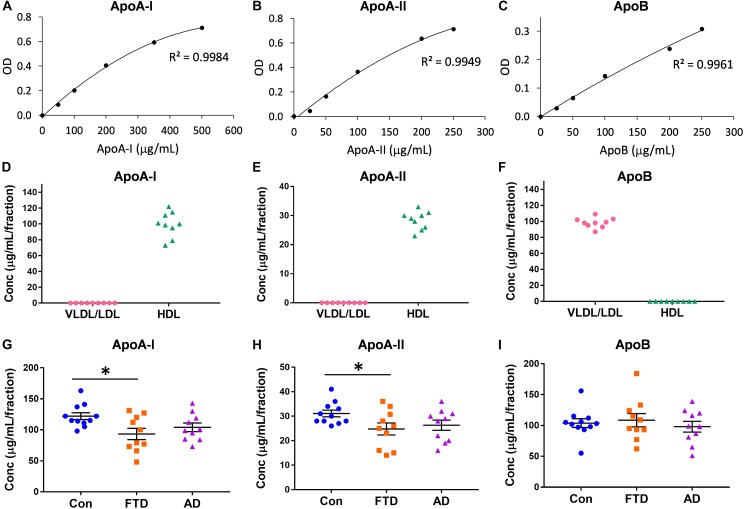
Apolipoprotein levels in lipoproteins in bvFTD, AD, and controls. **(A–C)** Apolipoprotein assay standard curves with *R*^2^ value. **(D–F)** Confirmation that apoA-I and apoA-II are present only in HDLs and apoB is present only in VLDLs/LDLs. **(G–I)** Concentrations of apolipoproteins in bvFTD, AD, and controls. Bars represent mean and SE, ^∗^*p* < 0.05.

Virtually nothing is known about the distribution of apolipoproteins in HDLs and LDLs in bvFTD despite the fact that lipoprotein lipids are altered in bvFTD (Ahmed et al., 2014b; Kim et al., 2018). Here, we measured the concentrations of the major apolipoproteins carried by HDLs (i.e., apoA-I and apoA-II) and VLDLs/LDLs (i.e., apoB). Firstly, we showed that the assay standards were extremely accurate with *R*^2^ ≥ 0.9949 (Figures 2A–C). Secondly, we confirmed that apoA-I and apoA-II were present only in HDLs, and that apoB was present only in VLDLs/LDLs (Figures 2D–F). We then measured the concentrations of the three apolipoproteins in bvFTD, AD and controls, and analyzed the data using multivariate tests covarying for age and gender; neither age nor gender had any effect on the apolipoprotein concentrations. We found that both apoA-I and apoA-II concentrations were significantly decreased in bvFTD compared to controls (Figures 2G,H). There were no significant changes in apoB concentrations in either bvFTD or AD (Figure 2I).

### Assessment of Lipids Carried by Lipoproteins in bvFTD and AD

**FIGURE 3:**
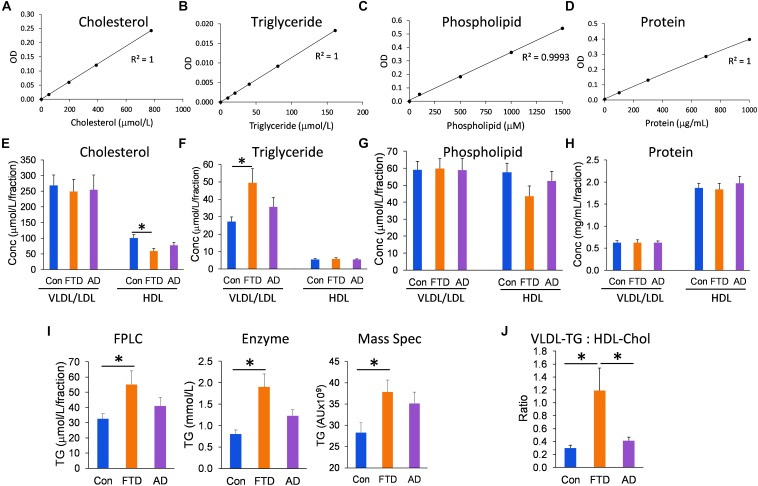
Lipid and protein levels in lipoproteins in bvFTD, AD, and controls. **(A–D)** Lipid and protein assay standard plots with *R*^2^ value. **(E–H)** Concentrations of lipids and protein in HDLs and VLDLs/LDLs in bvFTD, AD, and controls. **(I)** A comparison of total triglyceride measurements using FPLC, enzyme-based assay, and mass spectrometry. **(J)** The VLDL-triglyceride:HDL-cholesterol ratio is significantly increased in bvFTD compared to AD and controls. Data represent mean and SE as error bars, ^∗^*p* < 0.05.

Since the distribution of lipids in LDLs and HDLs is an integral part of lipoprotein metabolism, we measured the concentrations of all three major lipids present in lipoproteins – cholesterol, triglycerides, and phospholipids – in the VLDL/LDL and HDL fractions collected from each individual. We analyzed the data using multivariate tests covarying for age and gender, and calculated the mean for control, bvFTD and AD groups; age and gender had no significant effect on the lipid concentrations. Firstly, we showed that the assay standards were extremely accurate with *R*^2^ ≥ 0.9993 (Figures 3A–D). We then showed that HDL-cholesterol was significantly decreased in bvFTD compared to controls; no change in LDL-cholesterol (Figure 3E). VLDL-triglyceride was significantly increased in bvFTD compared to controls (Figure 3F). There was no change in HDL-triglyceride (Figure 3F). There were no significant changes in either VLDL/LDL- or HDL-phospholipid in bvFTD compared to controls (Figure 3G). There were no significant changes in either VLDL/LDL- or HDL-protein in bvFTD compared to controls (Figure 3H). These results confirmed manifestation of hypertriglyceridemia and hypoalphalipoproteinemia in bvFTD. We also showed that our measurements of total triglyceride using FPLC were consistent with two previous measurements using different methods (Ahmed et al., 2014b; Kim et al., 2018; Figure 3I). Finally, we calculated the ratio of VLDL-triglyceride to HDL-cholesterol and demonstrated that the ratio is significantly increased in bvFTD compared to both AD and controls (Figure 3J). This measurement could be considered for development as a potential biomarker to differentiate bvFTD from AD and controls.

### Increased Risk for Cardiovascular Disease in bvFTD

**FIGURE 4:**
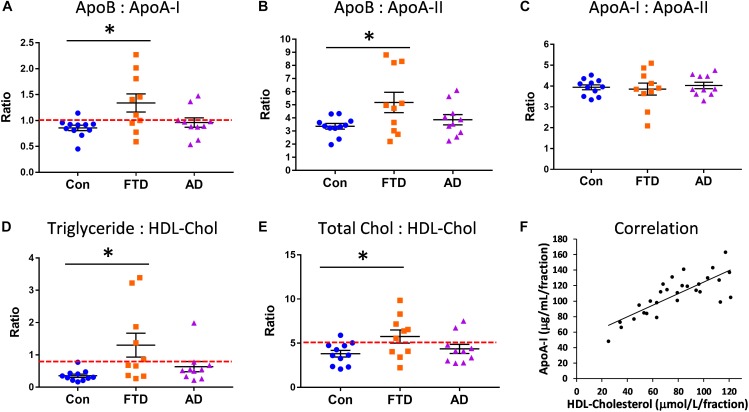
Increased risk for cardiovascular disease (CVD) in bvFTD. **(A)** The apoB:apoA-I ratio was significantly increased in bvFTD; a ratio value greater than 1.0 (red line) is regarded as increased CVD risk. **(B)** The apoB:apoA-II ratio was significantly increased in bvFTD. **(C)** The apoA-I:apoA-II ratio was unchanged in bvFTD and AD. **(D)** The triglyceride:HDL-cholesterol ratio was significantly increased in bvFTD; a ratio value greater than 0.87 (red line) is regarded as increased CVD risk. **(E)** The total cholesterol:HDL-cholesterol ratio was significantly increased in bvFTD; a ratio value greater than 5.0 (red line) is regarded as increased CVD risk. Bars represent mean and SE, ^∗^*p* < 0.05. **(F)** ApoA-I levels correlated positively with HDL-cholesterol levels.

Since our lipid and apolipoprotein results indicated manifestation of hypertriglyceridemia and hypoalphalipoproteinemia in bvFTD, we were interested in whether bvFTD had elevated risk for CVD. We calculated the ratios of apoA-I, apoA-II, and apoB in bvFTD, AD, and controls. The apoB:apoA-I ratio is known to be a robust indicator of CVD risk (Walldius and Jungner, 2006; McQueen et al., 2008). We found that the apoB:apoA-I ratio was significantly increased only in bvFTD (Figure 4A). Around 60% of bvFTD patients had a ratio value greater than 1.0 compared to only 20% for AD patients and 9% for controls (Figure 4A).

We found that the apoB:apoA-II ratio was also significantly increased in bvFTD (Figure 4B). Based on these results, both apoB:apoA-I and apoB:apoA-II ratios could be used to differentiate bvFTD from AD and controls in future biomarker studies. There were no significant changes in the apoA-I:apoA-II ratio in either bvFTD nor AD (Figure 4C). We also calculated two lipid ratios that are commonly used for determining CVD risk – (i) triglyceride:HDL-cholesterol and (ii) total cholesterol:HDL-cholesterol. In these two measurements ratios greater than 0.87 and 5.0, respectively, are regarded as increased CVD risk. We found that both ratios were significantly increased only in bvFTD (Figures 4D,E), once again indicating an increased CVD risk in bvFTD.

### Correlations

We also conducted correlation studies to test for any association between apolipoproteins and lipids. ApoA-I was positively correlated with HDL-cholesterol (*r* = 0.677, *p* = 2.9 × 10^−5^) (Figure 4F) and HDL-phospholipid (*r* = 0.713, *p* = 6.8 × 10^−6^), and negatively correlated with LDL-triglyceride (*r* = −0.568, *p* = 8.5 × 10^−4^). ApoA-II was positively correlated with HDL-cholesterol (*r* = 0.672, *p* = 3.5 × 10^−5^) and HDL-phospholipid (*r* = 0.778, *p* = 2.6 × 10^−7^), and negatively correlated with VLDL-triglyceride (*r* = −0.476, *p* = 6.7 × 10^−3^). ApoB was positively correlated with LDL-cholesterol (*r* = 0.805, *p* = 4.7 × 10^−8^) and LDL-phospholipid (*r* = 0.722, *p* = 4.5 × 10^−6^). ApoA-I was strongly correlated with apoA-II (*r* = 0.879, *p* = 8.1 × 10^−11^) as expected.

### Assessment of Plasma apoA-I Levels in bvFTD and AD

**FIGURE 5:**
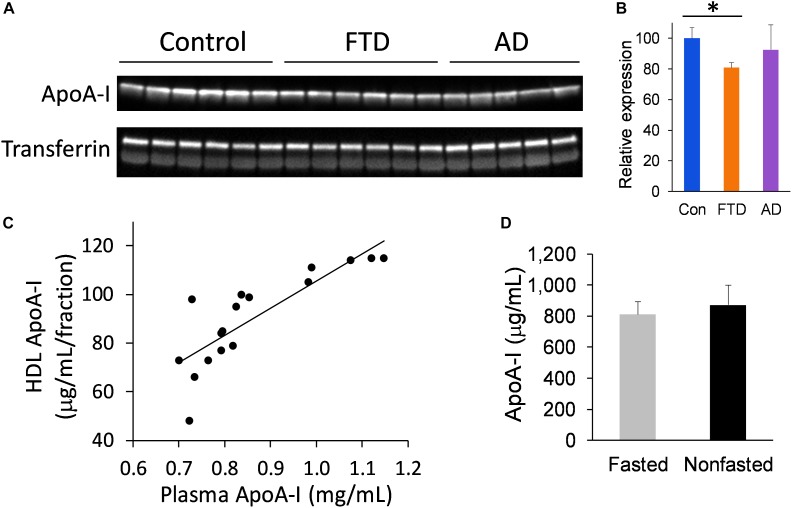
ApoA-I levels in whole plasma in bvFTD, AD, and controls. **(A)** Western blotting of plasma apoA-I; transferrin was used as a loading control. **(B)** Relative optical density of protein bands. Data represent mean (*n* = 6, 5, 6, respectively) and SE as error bars, ^∗^*p* < 0.05. **(C)** HDL fraction apoA-I levels correlated positively with whole plasma apoA-I levels. **(D)** No significant difference between fasted and non-fasted apoA-I levels in whole plasma.

We extended our study to assess the level of apoA-I in whole plasma to verify the findings from the FPLC fraction studies. Furthermore, analysis of apoA-I in whole plasma circumvents the need for plasma fractionation and therefore presents a simpler option for measuring apoA-I. We measured apoA-I levels in whole plasma from bvFTD, AD and controls, and found that apoA-I levels were significantly decreased in bvFTD compared to controls (Figures 5A,B), consistent with the fraction measurements. Moreover, apoA-I levels in the FPLC fractions correlated strongly with whole plasma apoA-I levels (Figure 5C). We also compared apoA-I levels in fasted and non-fasted whole plasma samples and found no significant difference (Figure 5D), indicating that apoA-I levels are not directly affected by food intake, as expected.

### Assessment of HDL Proteins in bvFTD and AD

**FIGURE 6:**
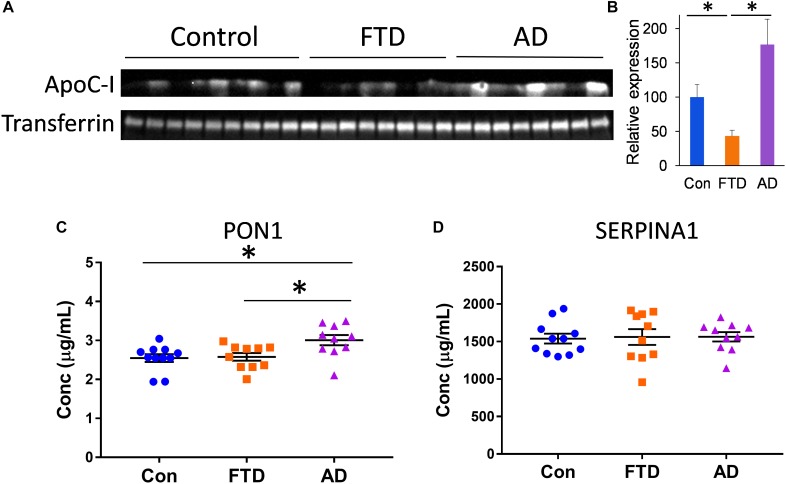
ApoC-I, PON1, and SERPINA1 levels in whole plasma in bvFTD, AD, and controls. **(A)** Western blotting of plasma apoC-I; transferrin was used as a loading control. **(B)** Relative optical density of protein bands; data represent mean and SE as error bars, ^∗^*p* < 0.05. **(C)** PON1 levels were significantly increased in AD compared to both bvFTD and controls as measured by ELISA; bars represent mean and SE, ^∗^*p* < 0.05. **(D)** SERPINA1 levels were unaltered in both bvFTD and AD compared to controls as measured by ELISA.

To further identify proteins that are different in bvFTD and AD, we assessed three other proteins that are carried by HDLs – apolipoprotein C-I (apoC-I), paraoxonase 1 (PON1), and serpin family A member 1 (SERPINA1). These proteins are intrinsically associated with HDL metabolism (Oliveira et al., 2004; Dumont et al., 2005; Inouye et al., 2012). We measured apoC-I, PON1, and SERPINA1 levels in whole plasma from bvFTD, AD, and controls. We found that apoC-I levels were significantly decreased in bvFTD compared to both AD and controls (Figures 6A,B), PON1 levels were significantly increased in AD compared to both bvFTD and controls (Figure 6C), and SERPINA1 levels were unaltered in both bvFTD and AD compared to controls (Figure 6D). ApoC-I could be considered for biomarker development for bvFTD.

## Discussion

Despite the fact that dyslipidemia underlies bvFTD, little is known about biochemical changes in lipoproteins in bvFTD. Here, we assessed apolipoproteins and lipids contained in HDLs and LDLs from bvFTD in comparison to AD patients and controls without neurological or psychiatric disorders. The aims of this study were to better understand the changes in lipoproteins in bvFTD, and to identify biomolecules carried by lipoproteins that could be considered for biomarker development in future studies. Previous studies have utilized whole plasma to analyze differences in lipid levels in bvFTD compared to AD and controls (Ahmed et al., 2014b; Kim et al., 2018). Here, we fractionated plasmas and analyzed lipids in individual lipoproteins, providing far more information on lipid changes in blood. Knowing in which lipoprotein particle lipid changes occur is extremely important as it allows a more accurate understanding of lipid dysregulation underlying bvFTD and consequently can lead to a more defined therapeutic strategy. In this study, we assessed all three major lipids in lipoproteins (cholesterol, triglycerides, and phospholipids) and showed that VLDL-triglyceride levels were significantly increased in bvFTD compared to controls. None of the three lipids in HDL and LDL were significantly altered in AD compared to controls.

Apolipoproteins carried by lipoprotein particles are integral to the structure and function of lipoprotein. Unlike AD, very little is known about changes in apolipoprotein levels in bvFTD. Apolipoproteins carried by HDL are known to be important in AD pathogenesis (Hottman et al., 2014). In this study, we showed that both apoA-I and apoA-II (carried by HDL) levels were decreased in bvFTD compared to controls. They were decreased in conjunction with decreases in HDL-cholesterol levels, as demonstrated by the strong positive correlation between ApoA-I/ApoA-II and HDL-cholesterol. These lipid and lipoprotein results clearly demonstrate that redistribution/remodeling of HDL had occurred in bvFTD, suggesting perturbation of HDL biology in bvFTD.

Since changes in diet and sedentary lifestyle, along with dyslipidemia, are prominent traits of bvFTD, we were interested in CVD status of bvFTD. We calculated three ratios – apoB:apoA-I, triglyceride:HDL-cholesterol, and total cholesterol:HDL-cholesterol – to determine CVD risk. All three measurements indicated increased CVD risk in bvFTD, in contrast to AD. The two lipid ratio measurements are based on the fact that HDL-cholesterol is inversely associated with CVD risk (Emerging Risk Factors Collaboration et al., 2009), and these measurements have been commonly used for assessing CVD risk for the past few decades. The apoB:apoA-I measurement is relatively new and is regarded as a stronger indicator of CVD risk than the triglyceride:HDL-cholesterol and total cholesterol:HDL-cholesterol ratios (Sniderman et al., 2003; McQueen et al., 2008). A significant advantage of this measurement over others is that non-fasted blood can be used, since protein levels are not directly affected by recent food intake (McQueen et al., 2008). Indeed, we clearly demonstrated that there were no significant differences between fasted and non-fasted plasma apoA-I levels. Another advantage is that apoB:apoA-I measurements can be attained directly from whole plasma without the requirement for plasma fractionation.

Although there are clear differences between CVD and neurodegenerative diseases, our data along with many others indicate that risk factors for CVD overlap with those for neurodegenerative diseases. Furthermore, growing data indicates that lipids and lipoproteins link CVD with neurodegenerative diseases. Cholesterol is central to CVD and it is becoming increasingly apparent that cholesterol is also extremely important to brain function as we know that almost a quarter of the body’s cholesterol is localized in the brain. It is a predominant component of the membranes of neurons and astrocytes, and of the myelin sheath of oligodendrocytes (Jurevics and Morell, 1995). We also know that lipoprotein complexes are vital for neuron function, including synaptic maturation and plasticity, and neurite outgrowth (Koudinov and Koudinova, 2001; Mauch et al., 2001).

We also assessed apoC-I, PON1, and SERPINA1 levels with the aim of further understanding HDL dysregulation in bvFTD and to identify potential biomarkers for bvFTD. We showed that apoC-I and PON1 levels were significantly altered in bvFTD and AD, respectively, compared to controls. ApoC-I regulates activities of several proteins involved in HDL metabolism, including lecithin-cholesterol acyltransferase and cholesteryl ester transfer protein. ApoC-I polymorphisms have also been identified as a risk factor for AD and CVD (Zhou et al., 2014; Allen et al., 2016; Chen et al., 2016). Very little is known about apoC-I in the context of bvFTD, other than that there are differences in linkage disequilibrium at the 19q13-q13.2 chromosomal region between bvFTD and primary progressive aphasia (Seripa et al., 2012), which is another subtype of FTD. ApoC-I plasma levels could be considered as a potential biomarker to differentiate bvFTD from AD and controls following further validation studies.

Paraoxonase 1 is an enzyme that hydrolyses organophosphate chemicals (pesticides) that are commonly used in agriculture. In addition to its detoxifying role, PON1 plays a role in protecting HDLs from oxidative damage and lipid peroxidation, though not consistent in all studies (Garner et al., 1998; Bhattacharyya et al., 2008; Mastorikou et al., 2008; Zerrad-Saadi et al., 2009; Besler et al., 2011). Currently, there is no consensus on the relationship between PON1 activity and AD risk, with some studies indicating that low PON1 activity is associated with an increased AD risk (Alam et al., 2014), whereas others indicate no association (Pi et al., 2012). Few, but not all, studies indicated that PON1 polymorphisms are associated with AD (Li et al., 2003; Leduc et al., 2009). Although PON1 has not been associated with bvFTD, it has been linked to amyotrophic lateral sclerosis (ALS) (Ticozzi et al., 2010), which is in the same disease continuum with FTD. Furthermore, pesticide toxicity has been shown to be implicated as a risk factor for ALS (Sutedja et al., 2009). PON1 could be considered as a potential biomarker to differentiate bvFTD from ALS following further validation studies. There were no significant changes in SERPINA1 levels in either bvFTD or AD compared to controls. SERPINA1 (also called alpha-1-antitrypsin) is a protease inhibitor that targets trypsin and elastase. In a recent gene-based association study, SERPINA1 was shown to be associated with progressive non-fluent aphasia (PNFA, a subtype of FTD), but not bvFTD (Mishra et al., 2017). Future studies could include testing whether SERPINA1 can differentiate bvFTD from PNFA.

We have demonstrated that significant biochemical changes occur in lipoproteins in bvFTD. We have also identified biomolecules and their ratios that could be considered for biomarker development for bvFTD. Future work would involve validating our findings in a larger cohort of bvFTD patients, particularly at preclinical and various stages of disease progression. This study represents the first analysis of apolipoproteins in bvFTD and has provided new insights into altered HDL function and elevated CVD risk in bvFTD.

## Author Contributions

WK conceived and designed the study. All authors participated in the acquisition and analysis of data. WK, K-AR, and GH drafted the manuscript.

## Conflict of Interest Statement

The authors declare that the research was conducted in the absence of any commercial or financial relationships that could be construed as a potential conflict of interest.
